# Associations of Gait Characteristics with Fall Risk and Frailty in Older Women

**DOI:** 10.1093/geroni/igaf122.3357

**Published:** 2025-12-31

**Authors:** Chitra Banarjee, Patria Marcano Maldonado, Md Sanzid Bin Hossain, Hwan Choi, Rui Xie, Ladda Thiamwong

**Affiliations:** University of Central Florida, Orlando, Florida, United States; University of Central Florida, Orlando, Florida, United States; University of Central Florida, Orlando, Florida, United States; University of Central Florida, Orlando, Florida, United States; University of Central Florida, Orlando, Florida, United States; University of Central Florida, Orlando, Florida, United States

## Abstract

Falls are the leading cause of injury-related death for older adults, aged 60 years and older. Because falls often occur during movement, it is crucial to understand dynamic balance. This preliminary work expands the standard Timed Up and Go (TUG) test of dynamic balance by integrating plantar insole sensors to administer the instrumented TUG (iTUG). We aim to identify the relationship between objective data from the iTUG with subjective measures. Gait characteristics consisted of stride velocity, center of pressure (COP) velocity, and single support phase (SSP) duration, and subjective measures were fall risk and frailty, evaluated by questionnaires. The fall risk was assessed using the Center for Disease Control and Injury Prevention (CDC) Stopping Elderly Accidents, Deaths and Injuries (STEADI) screening instrument, and frailty was assessed using Simple Frailty Questionnaire (FRAIL). The study included 13 older women (Mage=74.66±1.48 years) who first completed the standard TUG followed by three iTUG trials. Spearman’s correlations were used to evaluate associations between gait characteristics and subjective measures. A paired t-test was used to confirm the validity of the iTUG by comparing the average duration of the iTUG trials with the experimenter-recorded duration (p < 0.001). Greater fall risk was correlated with decreased stride velocity (p = 0.011), decreased SSP (p = 0.011), and increased COP velocity (p = 0.002). Increased frailty was correlated with decreased stride velocity (p = 0.005), but did not show correlations with SSP and COP velocity. These preliminary results specify dynamic balance deficits in older women showing that greater perceived fall risk and frailty coincides with cautious and unstable gait.

